# Adolescent idiopathic scoliosis with concomitant spondylolysis: choice of fusion levels and evaluation of the outcomes obtained leaving the lytic level not instrumented

**DOI:** 10.1007/s43390-023-00715-9

**Published:** 2023-06-21

**Authors:** Alice Baroncini, Antony Field, Anand H. Segar, Cheuk Bun Tse, Aleksandar Sevic, Haemish Crawford

**Affiliations:** 1 Department of Orthopaedics, Starship Hospital, Auckland, New Zealand; 2 Department of Orthopaedics and Trauma Surgery, RWTH Aachen University Clinic, Aachen, Germany; 3 Faculty of Medical and Health Sciences, University of Auckland, Auckland, New Zealand

**Keywords:** Adolescent idiopathic scoliosis, Spondylolysis, Spondylolisthesis, Fusion levels, Lowest instrumented vertebra

## Abstract

**Purpose:**

7% of adolescent idiopathic scoliosis (AIS) patients also present with a pars defect. To date, there are no available data on the results of fusion ending proximal to a spondylolysis in the setting of AIS. The aim of this study was to analyze the outcomes of posterior spinal fusion (PSF) in this patient cohort, to investigate if maintaining the lytic segment unfused represents a safe option.

**Methods:**

Retrospective review of all patients who received PSF for AIS, presented with a spondylolysis or spondylolisthesis and had a min. 2-year follow-up. Demographic data, instrumented levels, and preoperative radiographic data were collected. Mechanical complications, coronal or sagittal parameters, amount of slippage, and pain levels were evaluated.

**Results:**

Data from 22 patients were available (age 14.4 ± 2.5 years), 18 Lenke 1–2 and 4 Lenke 3–6. 5 patients (24%) had an isthmic spondylolisthesis, all Meyerding I. The mean preoperative Cobb angle of the instrumented curves was 58 ± 13°. For 18 patients, the lowest instrumented vertebra (LIV) was the last touched vertebra (LTV); for 2, LIV was distal to the LTV; for 2, LIV was one level proximal to the LTV. The number of segments between the LIV and the lytic vertebra ranged from 1 to 6. At the last follow-up, no complications were observed. The residual curve below the instrumentation measured 8.5 ± 6.4°, the lordosis below the instrumented levels was 51.4 ± 13°. The magnitude of the isthmic spondylolisthesis remained constant for all included patients. Three patients reported minimal occasional low back pain.

**Conclusion:**

The LTV can be safely used as LIV when performing PSF for the management of AIS in patients with L5 spondylolysis.

## Introduction

Posterior spinal fusion (PSF) currently represents the gold standard for the treatment of adolescent idiopathic scoliosis (AIS) [1]. The choice of the correct fusion levels is fundamental to guarantee the success of the surgical management and many studies have been conducted to investigate this aspect [2–5]. In patients with lumbar curves, in particular, the aim is to obtain a stable construct while maintaining the fusion as short as possible to limit the reduction in range of motion and the risk of adjacent segment degeneration [6, 7].

A particular challenge is represented by AIS patients who also present with a spondylolysis and/or an isthmic spondylolisthesis, who represent up to 7% of the AIS population [8]. In the setting of adult deformity surgery, the combination of a lumbar curve with spondylolisthesis can be managed with a long fusion including the pelvis. This avoids ending the construct above an unstable segment, and thus limits the risk of distal segment degeneration. When managing adolescent patients with a similar presentation surgeons rarely consider including the lytic level in the fusion, but alternative management options are represented by lysis repair or isolated fusion of the lytic segment. However, these options could lead to a more rapid degeneration of the unfused discs between the instrumented levels, low back pain or revision surgery.

Data regarding the long-term results of fusion ending proximal to a pars defect in the setting of AIS are scarce [9]. The aim of this study was to analyze the outcomes of posterior spinal fusion in patients who presented with AIS and a spondylolysis and/or isthmic spondylolisthesis, to investigate if maintaining the lytic segment unfused indeed represents a safe and stable option for the surgical management of these patients.

## Materials and methods

This retrospective study, level of evidence II, was conducted following the Strengthening the Reporting of Observational Studies in Epidemiology (STROBE) statement [10]. IRB approval was granted by the local authority.

### Patient selection

All patients who underwent posterior spinal fusion at our institution between 2010 and 2020 were screened for inclusion. The inclusion criteria were a diagnosis of AIS with concomitant spondylolysis and/or isthmic spondylolisthesis. Patients who did not have a minimum 2-year follow-up and who did not have a complete imaging (whole spine PA and lateral, side-bending or traction x-rays) preoperatively, at the first standing X-ray, the 6-week follow-up and at the last available follow-up were excluded from the analysis.

The diagnosis of spondylolysis and/or isthmic spondylolisthesis was based on X-rays only, as CT scans are not routinely performed prior to surgery. As this may lead to an underestimation of the number of patients with a spondylolysis, the records of patients who underwent revision surgery for mechanical complications (screw loosening, adjacent segment disease, distal junction kyphosis) and, thus, had a later CT were also screened for a previously undetected spondylolysis and/or isthmic spondylolisthesis to prove the internal validity of the collected data.

### Data extraction

The hospital medical records of the patients who met the inclusion criteria were screened by two experienced spine surgeons. Disagreements were discussed with the senior author until unanimous consensus was achieved. Demographic data (age, gender) and radiographic data (Risser stage, type of curve, Cobb angle, extent of the instrumentation) were collected. In particular, the upper and lower instrumented vertebra (UIV and LIV) were recorded along with the number of unfused discs between the LIV and the level presenting with a spondylolysis or spondylolisthesis. In the case of spondylolisthesis, the Meyerding grade was also recorded. For each patient, the end, neutral, last touched and stable vertebrae were identified to highlight the decision-making process in LIV selection.

### Outcomes of interest

The rate and type of mechanical complications was analyzed in the subset of patients who presented with a spondylolysis and/or spondylolisthesis. For patients who underwent revision surgery for mechanical complications, the rate of patients with spondylolysis and/or spondylolisthesis was recorded.

From a radiographic perspective, sagittal parameters (lumbar lordosis, LL, and pelvic incidence, PI) were collected for all patients. Furthermore, different measurements were performed to highlight any direct or indirect sign of distal segment degeneration. First, the magnitude of the residual curve below the LIV in the coronal plane and the lordosis between the lower endplate of the LIV and S1 were measured at the 6-week follow-up and at the last available follow-up. Second, the composite radiographic score (CRS) [11] was evaluated before surgery and at the last follow-up. The CRS score summarizes five radiographic indicators of disc disease (osteophytes, Schmorl’s nodes, intradiscal calcifications, sclerosis, endplate shape) and was employed as a direct assessment of disc degeneration. Lastly, any change in the Meyerding grade of the lytic vertebra was recorded. The 6-week X-rays were chosen over the first standing X-rays as the latter would have overestimated the magnitude of the residual curve and underestimated the amount of lordosis.

The medical records from the preoperative assessment and the last available follow-up were screened to investigate if the patients reported back or radicular pain and, if so, the frequency and intensity of the pain (visual analog scale—VAS) were noted.

### Statistical analysis

The statistical analysis was conducted using STATA/BE 17 (StataCorp, College Station, TX). For descriptive statistics, continuous data were expressed as mean ± standard deviation; discrete data were expressed as median and range, and qualitative data were expressed as percentages. Continuous variables were expressed as mean ± standard deviation and were compared with a *T* test.

## Results

### Patient recruitment

The records from 251 patients diagnosed with AIS and who underwent PSF between 2010 and 2020 were screened for inclusion. Of them, 22 patients presented with a spondylolysis and/or isthmic spondylolisthesis. One patient was excluded due to the lack of 2-year follow-up. Thus, data from 21 patients were available for the analysis (follow-up rate 95%). The mean follow-up for these patients was 41 ± 20 months.

Nine of the two hundred and fifty-one patients who underwent posterior fusion during the observation period require revision for mechanical complications (eight distal loosening, one non-union). On careful review of all the available imaging, none of these patients presented with a spondylolysis or a spondylolisthesis.

### Data extraction

The detailed demographic and radiographic data for each patient are presented in Table 1. All patients had an L5 spondylolysis. Five patients (24%) were male. The average age was 14.4 ± 2.5 years. The median Risser stage was 1, range 0–5. Four patients had a structural lumbar curve. Five patients (24%) had an isthmic spondylolisthesis, all Meyerding I. The mean preoperative Cobb angle of the instrumented curves was 58 ± 13°.

**Table 1:** Summary of the demographic and radiographic characteristics of the included patients

	Gender	Age	Risser grade	Cobb angle	Lenke type	Listhesis	Meyerding grade	Instrumented levels	LIV	N. of segments between LIV and lysis	CRS preOP	CRS last follow-up	Revision?
1	F	16	4	40	1	N		T3-L2	TV + 2	3	0	1	N
2	F	13	0	65	1	N		T4-L3	TV −1	2	0	0	N
3	F	13	0	59	1	N		T4-L3	TV −1	2	0	0	N
4	F	13	1	45	1	Y	1	T4-T11	TV	6	0	0	N
5	F	13	2	71	2	Y	1	T3-L3	TV	2	0	0	N
6	F	18	5	47	1	N		T3-L1	TV + 1	4	1	1	N
7	F	13	0	49	1	Y	1	T4-L2	TV	3	0	0	N
8	F	14	1	57	1	N		T3-T12	TV	5	0	0	N
9	F	12	0	64	1	N		T4-L2	TV	3	0	0	N
10	M	21	5	64	3	N		T4-L4	TV	1	0	1	N
11	F	16	4	40	5	N		T10-L3	TV	2	0	0	N
12	M	15	1	84	1	N		T4-L2	TV	3	0	0	N
13	F	12	0	81	1	Y	1	T4-L3	TV	2	0	0	N
14	F	11	0	73	5	N		T4-L4	TV	1	1	1	N
15	M	14	2	52	1	Y	1	T4-T12	TV	5	0	0	N
16	F	16	1	46	1	N		T3-T11	TV	6	0	1	N
17	F	13	1	70	2	N		T3-L2	TV	3	1	1	N
18	F	17	5	50	1	N		T4-L1	TV	4	0	0	N
19	M	11	2	60	1	N		T4-T12	TV	5	0	0	N
20	F	18	5	46	6	N		T4-L3	TV	2	0	1	N
21	M	14	2	57	2	N		T2-T12	TV	5	0	0	N

For double curves, the Cobb angle refers to the curve with greatest magnitude. The localization of the lytic vertebra was L5 for all patients. *LIV* lowest instrumented vertebra, *TV* touching vertebra, *CRS* composite radiographic score, *Y* yes, *N* no

For all but four patients, the LIV corresponded to the last touched vertebra (LTV), intended as the most caudal vertebra of the curve to be touched by the central sacral vertical line (CSVL). For two patients with a kyphosis at the thoracolumbar junction, the chosen LIV was distal to the TV (touching vertebra) to improve the sagittal profile (TV + 2 and TV + 1). For two patients, both with structural lumbar curves, the LIV was one level proximal to the LTV (LTV-1). The number of mobile discs between the LIV and the spondylolysis ranged from 1 to 6.

### Outcomes of interest

At the last available follow-up, none of the patient had required revision surgery or had experienced any other complication, mechanical or of other kind.

The mean PI was 44.4 ± 18.6°; the mean LL was 58.9 ± 13.2° before surgery and 57 ± 11.2° at the last follow-up, with a mean PI–LL value of −12.4 ± 10°. The residual curve below the instrumentation measured 7.5 ± 6.7° at the 6-weeks follow-up and 8.5 ± 6.4° at the last available follow-up (*P* = 0.3). Lordosis below the instrumented levels increased from 47.2 ± 11.3° to 51.4 ± 13° (*P* = 0.02). The magnitude of the isthmic spondylolisthesis remained constant for all included patients.

Prior to surgery, the CRS was 1 for three patients, in all cases irregularities of the endplate at L5/S1, and 0 for the remaining 18 patients. At the last available follow-up, the CRS was 1 for seven patients (endplate irregularities or moderate sclerosis at L5/S1) and 0 for the remaining 14.

Regarding pain assessment, six patients reported preoperative axial pain after sports or after standing or sitting for a long time. Pain intensity was evaluated as VAS 3 to 4 by all patients. None of the patients experienced radiculopathy. At the last follow-up, all patients were asked whether they experienced any pain. Only three patients reported occasional low back pain without radiculopathy or neurologic symptoms, all three had also reported back pain prior to surgery. The pain was reported as VAS 2 by two patients and VAS 4 by one patient, all of them had a thoracic instrumentation to T12 or L1. Thus, all presented six or five mobile discs, respectively, and five or four mobile segments between the LIV and the lytic vertebra. In patients not reporting postoperative pain, the number of free discs between the LIV and the lytic vertebra ranged from 1 to 6.

**Fig. 1:**
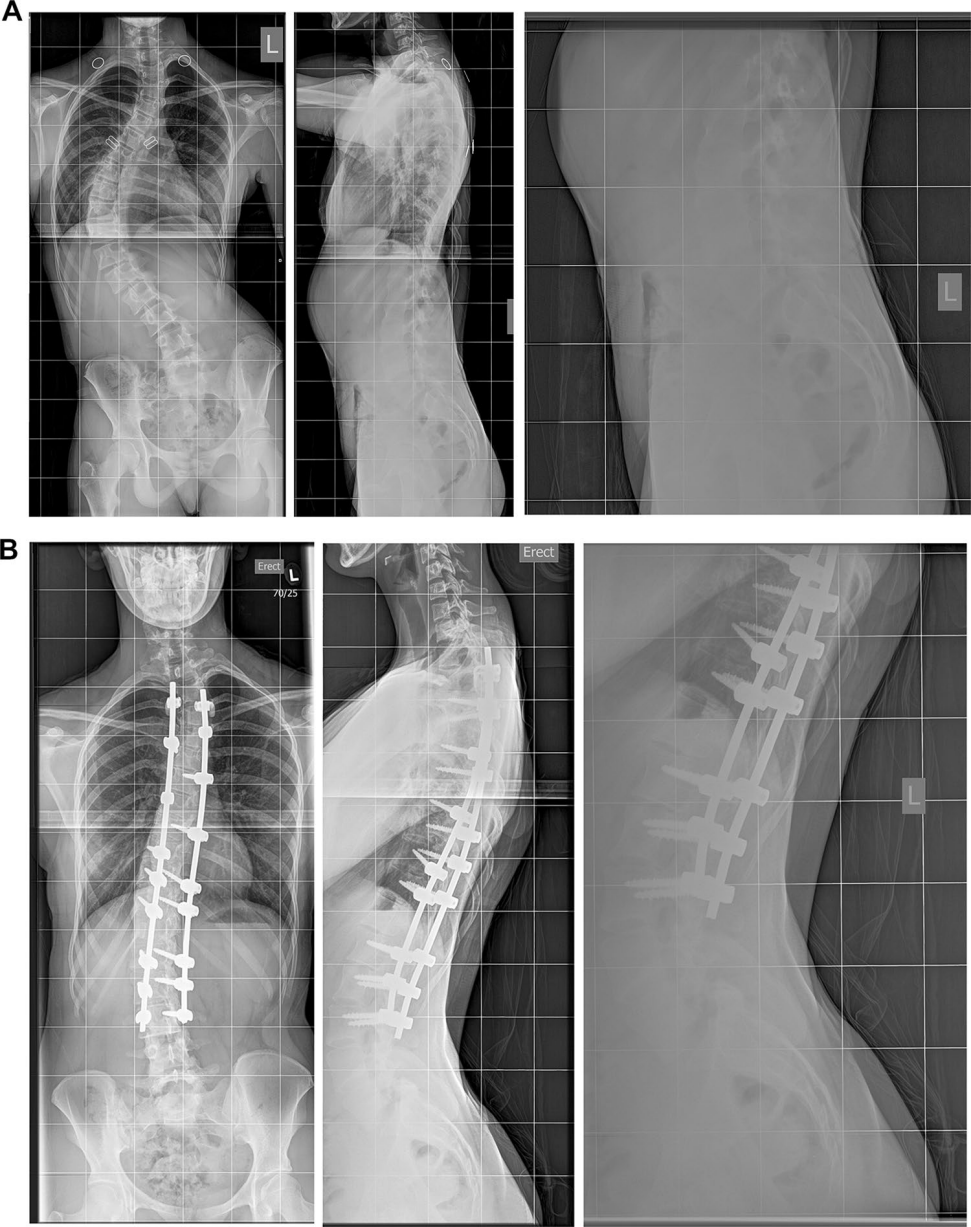
Clinical example of a 13-year-old girl with a 70° curve and a grade 1 isthmic spondylolisthesis (**A**). Four years after T3-L3 posterior fusion (LIV = EV), the X-ray showed a stable correction, there was no progression of the slippage and the patient was pain free (**B**)

A clinical example is shown in Fig. 1.

## Discussion

The main finding of this study is that leaving as few as one segment between the LIV and the level of the spondylolysis or spondylolisthesis was well tolerated and did not lead to painful symptoms, increased disc degeneration or further slip at a mean 40 months after surgery.

It is established that, after PSF, the levels distal to the LIV and in particular the L5/S1 discs are prone to degeneration [12]. While the choice of LIV may not affect distal disc degeneration, patients with a fusion to the low lumbar spine more often present with low back pain [6] and fusion to the low lumbar spine is associated with a delay in the return to the activities of daily living [7]. For these reasons, scoliosis surgeons aim to keep the instrumentation as short as possible and the TV is often chosen as the LIV, as it allows to obtain a stable construct while maximizing the number of uninstrumented vertebrae below the instrumentation [13–15]. However, patients with isthmic spondylolysis often present a vacuum phenomenon and a limited disc height at the affected level [16] and may, therefore, be at higher risk for disc degeneration. Some studies even reported these patients to have a poorer health-related quality of life than AIS patients [17]. Thus, it is questionable whether leaving a lytic level uninstrumented when performing PSF for AIS can guarantee stable and predictable results over time.

While some authors investigated the different options for surgical management of AIS patients with spondylolisthesis in the past [16, 17], these data cannot be transferred to the currently available instrumentations and surgical techniques. Only one recent study investigated the effects of fusion for AIS in patients with concomitant spondylolisthesis, observing that these patients obtained postoperative SRS-22 scores similar to those of the patients who did not present a spondylolisthesis [18]. Similar to the results presented in this cohort, patients with a low-grade spondylolisthesis did not present a slip grade progression or an increase in the amount of slippage [18].

Previous long-term studies on the outcomes of the Harrington instrumentation showed that a PI–LL mismatch is the single radiographic parameter associated with a reduced quality of life [19]. While the difference in type of instrumentation does not allow a direct comparison, it is reasonable to expect that a PI–LL mismatch would cause pain and disability in patients who were instrumented with modern pedicle screws constructs. On average, the presented cohort did not present a PI–LL mismatch, so that we do not expect this parameter to affect the patients’ quality of life, pain levels or slippage worsening. Furthermore, the presented cohort showed an averagely low PI, that is believed to be associated with a lower risk of slippage progression in comparison to patients with a high PI (> 60°) [20]. Further studies on a larger and more heterogeneous cohort will be required to investigate the effects of sagittal alignment on the progression of non-instrumented spondylolisthesis in patients who underwent fusion for AIS, and in particular in patients with high PI as these subjects might be at higher risk for progression.

In the present study, we observed only limited radiographic sign of distal segment degeneration. This is a relevant finding especially in patients with only one or two uninstrumented levels above the spondylolysis. The curve below the instrumentation remained stable over time both in the coronal and in the sagittal plane. While a statistically significant increase in the LL was observed, the difference was within the acceptable measurement error and is of minimal clinical significance. Furthermore, we did not observe an increase in the magnitude of the spondylolisthesis in any of the observed cases. The question rises, whether the follow-up of the present study would be sufficient to identify changes deriving from instability. While no data are available for the adolescent population, studies on adult surgery showed an average 52-months timespan between index surgery for revision due to adjacent segment instability [21, 22]. Considering the time intervening between symptoms onset, diagnostic and surgical planning, the average follow-up of 41 months should have been sufficient to observe at least initial changes. Regarding the CRS score, the data observed in the present study are in line with previously published findings in patients with AIS but no spondylolysis or spondylolisthesis [11]: both in Lonner’s and in the presented cohort, less than 20% of patient had a CRS = 1 before surgery, and about 30% of subjects had a CRS = 1 around the 5-year follow-up [11]. Five of the seven subjects with CRS = 1 had three or less uninstrumented segments below the instrumentation. This is coherent with the finding by Lonner et al. that instrumentations with more cephalad LIV are more prone to disc degeneration. Overall, given the similarities between the observed data and data obtained from the literature, degenerative changes might be linked more to the type of instrumentation and to the natural evolution of the segments distal to the instrumentation rather than to the presence of a spondylolysis or spondylolisthesis.

In an effort to maintain the mobility of the lumbar spine, surgeons have been increasingly performing selective thoracic fusion with positive results. Selective thoracic fusion might be even more relevant in the setting of AIS with spondylolisthesis or spondylolysis, as it would allow to maximize the uninstrumented levels between the LIV and the lytic vertebra. Only one patient in the presented cohort underwent selective thoracic fusion, so that data are too scarce to highlight possible differences in the outcomes of interest between this patient and those who received fusion into the lumbar spine.

While the number of the included patients is too small to reach a definitive conclusion on the topic, the high consistency among the observations strongly suggests that ending a PSF to the LTV is a safe strategy for AIS patients who also present with spondylolysis or isthmic spondylolisthesis. It is important to highlight, however, that none of the patients included in the present study presented with a high-degree spondylolisthesis (Meyerding Grade ≥ 3). This cohort may present with different biomechanical characteristics and our considerations cannot be directly extended to this population.

This study does not come without limitations, the first being represented by the retrospective nature of the analysis. Furthermore, the lack of a CT scan may have led to an underestimation of the number of patients with a spondylolysis from the initial cohort, as plain radiographs are at times insufficient to observe a pars defect without spondylolisthesis. We would expect that larger slips would be at greater risk of progression, and these are the easiest to identify on plain films. Further studies on a larger cohort are required to sub-analyze patients with structural lumbar curves (only four patients in our population), as the biomechanical interaction between lumbar curves and concomitant spondylolysis or isthmic spondylolisthesis still remains unknown. Larger studies will be also required to highlight possible outcome differences in patients with different number of unfused levels. Overall, ulterior studies with a longer follow-up will be required to investigate possible long-term changes and exclude the development of instability later in life. While patient-reported outcome measures such as the SRS score were not available for the present cohort, future studies should focus on the subjective perspective of the patients as well. It is also important to highlight that all observed patients were still young at the time of the final evaluation and the present study only investigated the evolution of the lytic segment in the early years following posterior fusion for AIS. It is, thus, possible that patients will develop symptoms later in life, and future studies should investigate whether time to symptoms development or slippage increase, pain levels, and amount of degeneration are influenced by the number of unfused segments between the LIV and the spondylolysis.

## Conclusion

In the presented cohort of 22 patients, the touching vertebra could be safely used as LIV when performing PSF for the management of AIS in patients with L5 spondylolysis or low-degree L5/S1 spondylolisthesis. This option allowed the surgeons to maximize the number of uninstrumented vertebrae below the construct and guaranteed stable results in the mid-term. However, studies on a larger cohort will be required to confirm these findings and further research should focus on the subset of patients with spondylolysis or spondylolisthesis below a structural lumbar curve, to highlight potential differences in the behavior of this type of deformity.

## Data Availability

Data can be made available upon reasonable request.
